# Prioritising Microbiology in Secondary Education Addresses Emerging Scientific‐Social‐Educational Challenges and Competency Needs

**DOI:** 10.1111/1751-7915.70224

**Published:** 2025-08-29

**Authors:** Lara Amorim, Conceição Santos, Kenneth Timmis

**Affiliations:** ^1^ Department of Biology, Faculty of Sciences University of Porto Porto Portugal; ^2^ IB2Lab‐LAQV‐REQUIMTE—Associated Laboratory for Green Chemistry of the Network of Chemistry and Technology Porto Portugal; ^3^ Institute of Microbiology, Technical University of Braunschweig Braunschweig Germany

**Keywords:** 21st century skills, critical thinking, global citizenship, microbiology education, scientific literacy, STEM competencies, sustainability and SDGs, UNESCO competences

## Abstract

This paper explores the potential of microbiology education at the secondary school level as a catalyst for enhancing both science, technology, engineering and mathematics (STEM) literacy and essential 21st‐century competencies. As an inherently interdisciplinary field, microbiology offers an effective platform to develop scientific and technological knowledge while cultivating broader competencies, such as emotional intelligence, creativity and critical thinking, as outlined by UNESCO (UNESCO, 2016). Scientific, societal and educational (SSE) challenges for the next decade are interlinked, and many of these trans‐dimensional issues require a solid foundation in biology and microbiology. Thus, research and education must evolve in step with these trends. Despite growing advocacy for microbiology in high school curricula, a significant gap remains in understanding how its teaching aligns with these broader developmental goals. This disconnection is particularly evident in Europe, where STEM engagement among youth remains low. As an experimental and accessible science, microbiology offers unique pedagogical strategies that address not only the cognitive dimensions of STEM but also the skills and attitudes needed for a complex, digital and interdependent world. By integrating microbiology into secondary education, teachers can bridge the gap between scientific literacy and future readiness, empowering students to build a generation capable of shaping a sustainable and innovative future. In this discourse, we describe examples of topics and strategies that could be implemented at the secondary education level, as well as at other educational levels, including university.

## Graphical abstract

This work highlights how microbiology education can empower secondary school students with critical and systems thinking, fostering STEM literacy, overcoming barriers and preparing informed, responsible global citizens for a sustainable, digital and interconnected world.

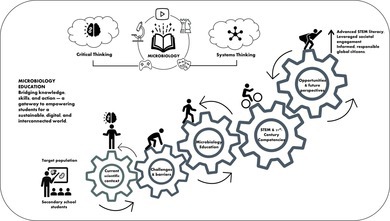

## 1 Microbiology at the Nexus of SSE Transformation: A Perspective for the Decade Ahead

The next decade will challenge humanity to address deeply interconnected scientific, societal, global and educational crises—from climate instability and antimicrobial resistance to misinformation and global health inequities. In this context, microbiology emerges not only as a scientific field of increasing strategic relevance but as a foundational lens through which we must view both biological complexity and social responsibility.

Our argument is simple: to confront the coming decade's trans‐dimensional challenges (Capozzi et al. 2012; OECD 2020a), we must urgently invest in microbiology literacy across all levels of society (Timmis et al. 2019). From primary education to advanced research, microbiology must be reframed as a tool for empowering citizens, informing policy and designing sustainable solutions. Yet, it remains underrepresented in many national curricula and undervalued outside of technical contexts.

### 1.1 Microbiology's Expanding Horizon: Where Science Meets Urgency

In today's world, microbiology stands at the intersection of scientific discovery and pressing global challenges. From the invisible yet powerful role of microbes in human/animal health, agriculture/farming and industry to their critical influence on climate change, pollution and biodiversity, microbiology touches nearly every aspect of our lives (e.g., see Anand et al. 2023). This section explores how microbiology is not just a field of study but a vital lens for addressing urgent scientific and social challenges (Table 1). Crucially, microbiology lies at the heart of the diverse dimensions of sustainability and the United Nations Sustainable Development Goals (United Nations 2015), as well as the strategies to achieve them.

**TABLE 1:** Examples of scientific and social challenges associated with microbiology.

Public Health and Microbial Literacy	Emotional and psychological impact of pandemics (e.g., COVID‐19) Importance of microbiology literacy for understanding: ○ Disease transmission ○ Antimicrobials and resistance ○ Vaccination ○ Hygiene and healthy living Rising rates of STIs (e.g., gonorrhoea, syphilis) among youth in Europe Declining vaccination rates and the resurgence of preventable diseases Obesity crisis Mental health crisis Misinformation and the role of social media in shaping health behaviours Vaccine hesitancy and public distrust
Microbiology in Society, Food and the Environment	Animal‐borne diseases (e.g., bird flu) affecting health, ecosystems and food security Food safety: understanding food labels and consumer awareness Bacterial threats to agriculture (e.g., *Xylella fastidiosa* ) and their impact on crop yields Overuse and misuse of antimicrobials in agriculture and farming and its global implications Insects as vectors of disease Understanding genetic engineering, synthetic biology, etc., and their ethical and public perception dimensions Microbial communication (e.g., quorum sensing) and environmental applications Microbes as food (single‐cell protein, edible microbes, microbial biomass) Pro‐, pre‐ and postbiotics; microbiome interventions
Educational Barriers in Microbiology Learning	Curriculum stagnation Curricular overload leaving insufficient time for in‐depth microbiology Teacher workload burden Lack of lab facilities and hands‐on opportunities Limited teacher training and confidence in microbiology instruction Restricted budgets limiting access to: ○ Laboratory consumables ○ Digital tools and simulations ○ Professional development
Challenges in Using Digital and AI Tools in Microbiology Education	Accuracy risks: AI may oversimplify or misrepresent microbiological concepts Hands‐on gap: Virtual labs cannot fully replace physical techniques like aseptic handling or staining Data bias: AI trained on skewed datasets may impact outbreak modelling or microbial identification Digital divide: Unequal access to internet and devices limits learning opportunities Loss of empathy and soft skills: Online learning may reduce opportunities for ethical discussion and interpersonal development Gamification overuse: Excessive use of game elements may distract from deep learning Privacy concerns: AI tracking raises ethical questions around student data and surveillance Cognitive skill reduction: Overreliance on AI tools may reduce students' development of critical reading, writing, comprehension, summarisation, analysis and deep‐thinking skills essential in microbiology Misuse of inadequate sources: Students may use unreliable websites or unvalidated AI platforms, leading to misconceptions or inaccurate microbiological knowledge Teacher and tutor challenges: Teachers face additional burdens to ensure students use digital tools ethically, critically and effectively, maintaining essential competencies without overdependence.
Ethical, Cultural and Structural Limitations	Insufficient ethical training in science curricula Insufficient training in critical thinking, systems thinking and the interconnectivity of things Limited interdisciplinary teaching and collaboration (e.g., integration with public health, ethics or policy) Narrow framing of microbiological priorities (e.g., focus only on pathogens, not ecosystems or equity) Students from underrepresented backgrounds may feel excluded or undervalued Underserved schools may lack access to: ○ Advanced lab work ○ Field experiences ○ Mentorship from culturally relevant role models Education often overlooks global justice issues linked to: ○ Microbial research ethics ○ Health policy disparities ○ Pharmaceutical access and equity

#### 1.1.1 Antimicrobial Resistance: Innovation Amid Escalation

Antimicrobial resistance (AMR) is no longer a forecasted problem—it is a current crisis. As resistance outpaces drug development, microbiology's role in uncovering novel antimicrobials and alternative therapies (e.g., bacteriophages, peptides) (Alisigwe et al. 2025; Min et al. 2024; Noor 2023) becomes indispensable (WHO 2022). But beyond research, we face a social challenge: how to communicate the urgency of AMR in a way that drives behavioural change (e.g., see Merz et al. 2023)—in prescribing, in agriculture and in everyday hygiene.

#### 1.1.2 Microbial Communities and Environmental Stewardship

Modern science increasingly recognises that living organisms—including humans and other animals and plants—coexist with complex microbial communities or microbiomes, which play essential roles in their health, development and adaptation changing environments. This integrated perspective has revolutionised fields such as precision medicine, veterinary care and sustainable agriculture (Crowther et al. 2024; Kuntz and Gilbert 2017; Zhou et al. 2024). Beyond these applications, microorganisms are now being strategically harnessed in targeted environmental bioremediation—a powerful yet underutilised biotechnology. By deploying pollutant‐degrading or transforming microbes in contaminated soils, water bodies and industrial sites, bioremediation enables the restoration of degraded ecosystems through eco‐friendly and sustainable means. This approach exemplifies how microbiological knowledge can be transformed into concrete, nature‐based solutions to global environmental challenges (Ruan et al. 2024).

#### 1.1.3 Public Health and Literacy in the Age of Misinformation

Understanding how diseases are transmitted within and between species is essential for managing future pandemics and ensuring effective public health responses. Microbiology literacy plays a crucial role in helping individuals grasp key issues such as infectious disease spread, hygiene practices, antibiotic resistance and the importance of vaccination. Without this foundational knowledge, misinformation can spread easily, vaccine hesitancy may increase and individual behaviours can unintentionally heighten societal health risks—for example, by hindering efforts to achieve herd immunity and the protection it offers vulnerable members of society. Educated citizens are better equipped to make informed, balanced decisions, promote hygienic habits and contribute to community resilience. This is particularly pressing given the documented rise in sexually transmitted infections (STIs) in Europe; the European Centre for Disease Prevention and Control (ECDC) reported continued increases in STI cases across the EU in 2023, with increases in gonorrhoea, syphilis and chlamydia (ECDC 2025a, 2025b, 2025c).

#### 1.1.4 Artificial Intelligence (AI): Microbiology's New Frontier

Though still in early stages, AI is already transforming microbiology by enabling the analysis of complex microbial datasets, enhancing the accuracy of pathogen identification and strengthening disease surveillance systems (Al‐Antari 2023; Shelke et al. 2023; Tripathi et al. 2024). Machine learning (ML) algorithms are being increasingly employed across research, clinical and industrial contexts to predict infectious disease outbreaks—including COVID‐19, tuberculosis and HIV infection—and to forecast antimicrobial resistance (AMR). As the interdisciplinary field of Digital Microbiology continues to emerge (Mohseni and Ghorbani 2024), there's a growing demand for professionals skilled in both microbiology and data science. So, integrating these innovations into early education is essential for preparing the next generation to navigate and contribute to a digital health landscape (Tsitou et al. 2024).

#### 1.1.5 Toward a Future of Rapid, In Situ Pathogen Diagnostics

The ability to detect pathogens quickly and accurately is becoming a cornerstone of modern healthcare, veterinary practice and agricultural biosecurity. Advances in molecular diagnostics—ranging from DNA‐based assays to antibody, peptide and spectral sensing technologies—are enabling faster, more sensitive and increasingly point‐of‐care solutions. These tools are not just scientific innovations; they represent critical infrastructure for real‐time, personalised medicine and more responsive public health strategies (Moore et al. 2023). As diagnostic platforms become more portable, affordable and user‐friendly, their integration into everyday settings—from clinics to farms—will reshape how we manage outbreaks and monitor microbial threats (Ahmed et al. 2014; Silva et al. 2024). They constitute vital enablers of increased patient participation in their own healthcare that will be important in countering otherwise diminishing access to healthcare as it becomes ever more expensive (Timmis and Timmis 2017).

#### 1.1.6 Microbial Engineering and the Rise of Synthetic Biology

Synthetic biology is redefining what it means to *engineer life*. No longer confined to natural capabilities, microbes can now be redesigned or entirely constructed to produce biofuels, pharmaceuticals, biodegradable materials and more (Noor 2023). At the heart of this transformation lies the concept of the *engineered chassis*—custom microbial platforms tailored for industrial or medical functions (Jones et al. 2024; Liu et al. 2020). Synthetic biology strategies and applications are increasingly enhanced by artificial intelligence (Yu et al. 2025). These breakthroughs promise powerful contributions to sustainability and innovation, but they also raise profound questions about biosafety, ethics and environmental impact. As synthetic biology moves from research to global markets, education must keep pace, preparing future professionals to navigate the technical, regulatory and societal dimensions of a world increasingly shaped by engineered microbes.

### 1.2 Educational Challenges for High School Microbiology Over the Next Decade

Microbiology offers high school students a gateway to understanding not only biological systems but also many of the major societal challenges of our time—from antimicrobial resistance and pandemics to sustainable agriculture and synthetic biology. Its inherently interdisciplinary nature positions it as a foundational science for future careers in biology, medicine, pharmacology, biotechnology and environmental science. But, as can be appreciated from the foregoing, knowledge of microbiology can also be important for careers in other, widely diverse sectors that include agriculture and veterinary science, food and beverages, chemistry, engineering (e.g., instrumentation and sensors), energy, resource mining and recovery, materials science, waste management, personal care products, laboratory supplies, innovation, investment and financial services, law enforcement (forensics), quality control, biodiversity, climate and meteorology, geology, history (microbes have seriously influenced the course of history), science journalism and, of course, education. If the appropriated topics are selected, microbiology has exceptional power to fascinate, inspire and propagate, not only among students but also the wider public (Timmis et al. 2020). It incentivises educational engagement and all of the developmental benefits that education entrains, and that have been discussed in this article. However, introducing microbiology meaningfully at the secondary level requires overcoming several key challenges over the coming decade—challenges that are simultaneously conceptual, infrastructural and pedagogical in nature (Table 2).

**TABLE 2:** Microbiology educational challenges and solutions.

Challenge	Educational solutions
Learner interest and engagement	Emphasise personal relevance of microbial activities for everyday life (e.g., body odour, fibre for health, the climate change footprint of ruminant feed animals; see Data S1 of Timmis et al. 2024, Supporting Information) Emphasise fascinating unique aspects of microbes, for example, related to space travel, glow (bioluminescence), eat plastic and degrade oil spills Student preparation of fermented foods Gamified learning tools
Keeping microbiology education current with scientific advances	Update curriculum themes: shift from a disease‐only focus to a holistic view that includes beneficial microbial roles Offer continuous professional development for educators Provide training and curated lists of reliable, accurate and recommended digital microbiology resources for effective teaching Connect content to current real‐world issues Encourage publishers to review and update textbooks in collaboration with experienced microbiologists to ensure accuracy and current information
Addressing conceptual complexity and student misconceptions	Use layered, scaffolded teaching approaches Incorporate relatable real‐world examples Provide hands‐on, inquiry‐based learning experiences Address common misconceptions directly through discussion, feedback and targeted student projects
Time constraints, limited resources and teacher preparedness	Advocate for curriculum reform to prioritise microbiology Provide targeted professional development Ensure equitable resource distribution Use innovative, low‐cost strategies (e.g., simulations, at‐home experiments, collaborative projects)
Countering misinformation and promoting scientific literacy	Use project‐based learning with socially relevant microbiology case studies Integrate media literacy: teach students how to evaluate sources, identify bias and detect misinformation Emphasise the link between microbiology literacy and public health outcomes, including sustainability
Embracing the digital transition: AI, e‐learning, and gamification	Employ adaptive learning platforms that personalise content difficulty and pace Use AI tutors or chatbots to simulate lab support or provide instant feedback Develop modular online courses or microlearning units (e.g., immunology, sterilisation) Integrate multimedia: animations, 3D models and lab videos Design microbiology games (e.g., outbreak simulations, microbiology‐themed puzzles) Utilise virtual reality to explore microbial environments (e.g., inside the human gut) Facilitate collaboration through online platforms (e.g., Padlet, Slack, Google Docs) Use digital peer‐review for project feedback Leverage mobile tools: flashcards, podcasts, daily microbiology quizzes
Broadening the scope: inclusion, interdisciplinarity and ethics	Implement a culturally responsive curriculum with global case studies Provide accessible and multilingual learning materials Integrate microbiology across disciplines (e.g., health, ethics, environmental science) Include bioethics modules and discussions Promote public engagement through Service‐Learning Use diverse group assignments with rotating roles to ensure inclusion

#### 1.2.1 Keeping Microbiology Education Current With Scientific Advances

Microbiology education must keep pace with rapidly evolving scientific discoveries. Topics such as antimicrobial resistance, microbiomes, synthetic biology and viral evolution are reshaping not only the life sciences but also public health and environmental policy. Updating curricular themes and empowering teachers with continuous professional development is critical—not only to reflect current science but to show students the relevance and applicability of microbiology in real‐world issues such as pandemics, sustainable agriculture and climate change.

#### 1.2.2 Addressing Conceptual Complexity and Student Misconceptions

Microbiology often involves abstract, invisible or interdisciplinary concepts that are challenging for students to grasp. Many microbial phenomena—such as viral replication, molecular biology, gene editing and immunological principles—require careful teaching strategies to balance accuracy with accessibility. Compounding this is the common misconception that microorganisms are only harmful—the problem of germophobia (Timmis et al. 2024), overshadowing their vital roles in ecosystems, human health and biotechnology. These issues can be addressed by introducing microbiology in increasingly complex layers (several examples are presented in Table 1), incorporating hands‐on activities and using real‐world examples to illustrate microbial benefits. Such approaches support sustained student engagement and more nuanced understanding of the microbial world.

#### 1.2.3 Time, Resources and Teacher Preparedness

Many teachers face time constraints due to content‐heavy curricula that leave little room to expand into microbiology topics (OECD 2020b; Petersen et al. 2020; Wang 2024). Others report limited confidence in teaching advanced microbiology due to a lack of training or the absence of laboratory facilities in their schools. Inadequate infrastructure—such as lack of funding for equipment—further restricts opportunities for hands‐on experimentation, which is crucial for deep understanding. This is compounded by educator task overload, which not infrequently leads to burnout, and educator loss from the system because of insufficient investment in education. Nevertheless, microbiology topics can still be meaningfully explored through simplified practical activities, virtual simulations, low‐cost experiments and class excursions (McGenity et al. 2020), allowing educators to introduce core ideas within existing curricular frameworks. It is, however, crucial to develop and offer educators microbiology teaching materials that excite both them and their pupils, to maximise teacher interest and satisfaction. This is also essential to maximising inclusivity of disadvantaged learners and supporting those with learning difficulties.

#### 1.2.4 Countering Misinformation and Promoting Scientific Literacy

The COVID‐19 pandemic has underscored the consequences of poor microbiology literacy, as misinformation about virus transmission, vaccine efficacy and public health interventions spread rapidly across social media. Anti‐vaccination movements and recent reports of increasing unvaccinated populations in Europe and the USA have heightened the risk of re‐emerging infectious diseases such as measles (Hamson et al. 2023; Hotez 2019). To counter these trends, schools must actively address social dimensions of microbiology in the classroom. This includes exploring the scientific basis of vaccination, hygiene and disease prevention, while also examining the spread and impact of misinformation. Teaching microbiology through real‐world, socially relevant case studies can help students better understand the implications of science for public health (Sagmeister et al. 2021; Timmis 2023b).

#### 1.2.5 Embracing the Digital Transition: AI, E‐Learning and Gamification in High School Microbiology Education

A pivotal challenge—and opportunity—for microbiology education in the coming decade is adapting to the rapid digitization of teaching and learning environments. The increasing use of digital platforms, artificial intelligence (AI) and gamified learning tools is reshaping how microbiology is taught at both high school and university levels (Apandi and Mokmin 2025; Del Cerro‐Sánchez et al. 2025; Rathod et al. 2024). This transformation goes beyond technological adaptation—it calls for a pedagogical shift that responds to the changing needs and habits of digitally native students. The COVID‐19 pandemic served as a catalyst for integrating virtual classrooms into mainstream education. Platforms such as Teams, Zoom, Google Classroom, Moodle, Panopto and Colibri became essential for content delivery and interaction (Aristovnik et al. 2023; DeCoito and Estaiteyeh 2022). These platforms now support flexible and interactive content delivery, especially when supplemented by digital resources such as microbiology videos and podcasts. For example, Zhang and Wasie (2024) proposed that *Information exchange platforms* (e.g., *WhatsApp, Facebook*) *break the traditional face‐to‐face communication model between schools, teachers and students, as well as promote interactions and bridge the communication gap with parents*. New gamified learning models are also proving effective for teaching microbiology in engaging and memorable ways (Hsiao et al. 2020; Onggirawan et al. 2023; Schweitzer et al. 2024; Zhang and Wasie 2024). As the use of AI grows in education, it will be critical to guide students in navigating both the possibilities and limitations of such tools—avoiding plagiarism while fostering digital literacy and independent thinking. Metaverse‐based tools and immersive learning environments are also beginning to emerge, opening new educational possibilities that must be balanced with concerns about data privacy, screen time and pedagogical efficacy (Agustini et al. 2023; Kim and Kim 2023; Martinez‐Garcia et al. 2023). By acknowledging and addressing these challenges, microbiology education in high schools can be transformed into a dynamic, relevant and empowering field of study. With appropriate support, innovation and policy alignment, educators can ensure that students not only learn about microbes—but are equipped to understand their significance in shaping our world.

#### 1.2.6 Broadening the Scope: Emerging Priorities for Inclusive and Engaged Microbiology Education

In addition to the above‐described challenges facing high school microbiology education, several emerging issues warrant attention. Equity and access remain critical concerns, as disparities in funding and infrastructure can limit opportunities for hands‐on learning and digital engagement in underserved schools. Moreover, integrating microbiology with other disciplines like environmental science and public health can enhance relevance and foster interdisciplinary thinking. As microbiological discoveries increasingly intersect with ethical and societal questions—such as the use of Clustered Regularly Interspaced Short Palindromic Repeats (CRISPR) or responses to antimicrobial resistance—it becomes essential to equip students with frameworks for ethical reasoning (Ejaz et al. 2025; Sjogren et al. 2023). Post‐pandemic classrooms also face the task of re‐engaging students, many of whom have experienced disruptions to practical science education. Class discussions about issues like global warming and planetary stewardship can promote awareness and engagement of young people who are typically motivated by sustainability issues. Finally, promoting inquiry‐based learning in environments with limited laboratory access calls for creative approaches to experimental teaching, ensuring that all students can experience the investigative nature of microbiology (Chiang et al. 2021; Spence et al. 2020; Timmis et al. 2024).

## 2 Microbiology Education: A Powerful Ally to Advance STEM Literacy and Societal Engagement

Although STEM education is usually discussed in relation to workplace skills and the needs of national economies, its concept is closely linked to scientific literacy, societal engagement and the Sustainable Development Goals. Microbiology aligns naturally with these priorities, offering interdisciplinary learning opportunities and real‐world applications. As emphasised by Amorim et al. (2025), Hansen et al. (2024) and Hilton and Hilton (2024), STEM education seeks not only to teach scientific content but to contextualise it within broader societal, environmental and technological frameworks. Microbiology supports this vision by bridging diverse fields—including genetics, molecular biology, immunology, biochemistry, and environmental science—and by promoting both technical and transversal skills (Lin and Tsai 2021). Integrating microbiology into STEM‐based strategies in formal, non‐formal and informal settings can develop essential scientific competencies, engage students with pressing global issues, and encourage pursuit of careers in biology, health sciences, biotechnology and related fields.

A central strength of microbiology education lies in its capacity to support integrative and complementary pedagogical approaches, such as hands‐on experiments, fieldwork, project‐based learning and contextualised teaching. These methods encourage critical thinking, problem‐solving and active participation—skills essential for scientific citizenship (Burks 2022; Fahnert 2017; McGenity et al. 2020; Pateman et al. 2025). Microbiology is also embedded in daily life, with obvious applications in health, food and environmental sustainability, helping students see its relevance beyond the classroom.

In fact, the field of microbiology is undergoing rapid and transformative advancements. Examples include the development of novel antimicrobials, microbiome‐based therapies and genome‐editing technologies like CRISPR. High‐profile scientific achievements in these areas—such as groundbreaking discoveries concerning nucleoside base modifications that enabled the development of effective mRNA vaccines against COVID‐19, recognised by Nobel Prizes awarded to Katalin Karikó and Drew Weissman—provide inspirational case studies that demonstrate the power of microbiological knowledge to shape the future. Indeed, some 43 Nobel Prizes have been awarded for discoveries in microbiology; new discoveries and applications are being made so rapidly that it was recently proposed that we have now entered the *Age of Microbial Technology* (Timmis and Hallsworth 2024). As such, microbiology is a nearly inexhaustible source of engaging and cutting‐edge content suitable for diverse educational levels, content that is reinforced by almost daily press reports.

Furthermore, microbiology fosters systems thinking and interdisciplinary integration, combining elements from biology, chemistry, mathematics and even physics, and ranging over medicine, agriculture, the environmental sciences, forensics, palaeontology, climatology, geology and indeed art, the social sciences, economics, law (Logan et al. 2025), etc. The connectivity and interdependencies of microbiology with other disciplines and human endeavours are truly astonishing (e.g., see Data S3 of Timmis et al. 2024, Supporting Information). This helps students develop scientific reasoning through real‐world applications—for example, understanding how basic biochemistry principles explain microbial metabolism or using mathematical models to analyse microbial population growth. The involved key competencies (e.g., systems thinking, interdisciplinary integration, scientific reasoning) not only build scientific literacy but also prepare students to engage with complex global challenges and the SDGs (Paz and Vasconcelos 2025; Romulo et al. 2024). Indeed, sustainability and the United Nations Sustainable Development Goals are readily woven into microbiology teaching resources, which then become educational instruments of sustainability awareness, responsible behaviour and solutions to problems (e.g., see Timmis et al. 2024).

Another important dimension is the emotional and social connection that microbiology can create. Its proximity to personal and public health, especially after the global COVID‐19 pandemic, makes it a subject with which students can readily identify. It provides both personal and collective relevance, allowing students to connect what they learn in school with real societal concerns, thereby enhancing motivation and the development of active, informed citizenship (Lloyd and Berry 2022; Sánchez‐Angulo et al. 2021). As an example, Service‐Learning, a methodology that promotes student learning while they provide a service to the community, allows students to work in real contexts and engage with societal needs (Gil‐Serna et al. 2025; Valderrama et al. 2025). Importantly, microbiology is a subject that readily interests and excites all age groups and, of relevance to education, teachers and their motivation.

Despite this potential, microbiology is still underrepresented in many secondary school programmes. Several authors have called for a stronger inclusion of microbiology in school science curricula (Davis et al. 2020; Ginnan and Bordenstein 2023; Sánchez‐Angulo et al. 2021; Timmis 2023a; Timmis et al. 2019). A more deliberate integration would not only enhance students' scientific understanding but also provide a clearer picture of how microorganisms influence virtually every aspect of life—from digestion and disease prevention to climate change and food production. It is also foundational to higher‐level studies in biology, biochemistry, pharmacology, medicine, agriculture, bioengineering and environmental sciences.

Importantly, microbiology is also a highly experimental and accessible science. Furthermore, being a highly experimental and interdisciplinary field, microbiology is rich in examples for simple experimental activities that can be reproduced in high schools with few or moderate resources. Many activities—such as culturing microbes from everyday surfaces, observing fermentation or investigating hand hygiene effectiveness using agar plates (Da Silva and De Lima 2020; Rezal et al. 2024; Scharfenberg and Marquardt 2015)—require minimal equipment and can be easily adapted to classroom settings, even in schools with limited resources. Introducing students to laboratory activities promotes skills in laboratory manipulation, experimental design and data analysis. Programs like the International Microbiology Literacy Initiative (IMiLI, Timmis et al. 2024), e‐Bug (Hayes et al. 2020), Tiny earth (Hurley et al. 2021), Microbes and You (Trudel et al. 2020) MicroMundo (Gil‐Serna et al. 2025) (or Methylothon Jones et al. 2022) provide structured, replicable experimental protocols and didactic materials tailored to secondary education. These initiatives aim to democratise microbiological education, ensuring that all students—regardless of geographic or socioeconomic background—can access high‐quality science education.

By providing opportunities to engage with real‐life questions and emerging scientific discoveries, microbiology supports the broader aims of STEM education: building literacy, curiosity and engagement (Kennedy and Odell 2014; Kent 2025). With thoughtful implementation, microbiology can be a driver of educational innovation, scientific empowerment and sustainable development, offering young learners the tools they need to navigate—and contribute to—a rapidly changing world (Fagunwa and Olanbiwoninu 2020; Timmis et al. 2024).

## 3 Microbiology Education as a Driver of Competences for the Future

Microbiology education at the secondary level offers a unique and underexplored opportunity to enhance both STEM literacy and key 21st‐century competencies. Despite increasing calls for the inclusion of microbiology in high school curricula (Davis et al. 2020; Ginnan and Bordenstein 2023; Sánchez‐Angulo et al. 2021; Timmis 2023a; Timmis et al. 2019)—given its highly interdisciplinary nature and its potential to enhance STEM‐related skills—there remains a lack of research exploring how the teaching of this science can promote not only scientific and technological cognitive abilities but also the broader *Competences for the Future* outlined by the United Nations Educational, Scientific and Cultural Organisation (UNESCO 2016) and others (e.g., United Nations 2022). This gap in the educational literature and practice has been noted by scholars (Fatton et al. 2021; Timmis 2023a). These competences encompass the development of knowledge, skills, attitudes, values and experiences that together form a comprehensive foundation for STEM education.

Moreover, there is limited understanding of the challenges teachers face in implementing these competencies and, ultimately, of how microbiology education may nurture students' interest and engagement in STEM fields. Recent indicators (European Schoolnet 2023; European Commission 2025) show that STEM disciplines in Europe still struggle to attract young people, prompting initiatives aimed at increasing students' motivation to pursue degrees in these areas.

In today's world—where sustainability, safety and technological advancement are increasingly interwoven—it is essential that both young people and adults develop skills that are critical to navigating daily life. In this context, the UNESCO (2016) identifies a set of key future‐oriented competencies, listed in descending order of frequency and relevance: emotional intelligence/empathy, creativity, critical thinking, collaboration, effective communication, complex problem solving, digital literacy, learning to learn, resilience/preservation, adaptability, conflict resolution and leadership.

### 3.1 The Role of Emotions and Emotional Intelligence in Microbiology Education

Emotions play a pivotal role in activating—or disengaging—student attention and, more critically, in shaping motivation to learn. The emotional state of a student during classroom engagement can significantly influence cognitive processing and knowledge retention. Positive emotions, such as curiosity, joy and empathy, tend to foster engagement and openness to new ideas, whereas negative emotions like anxiety, fear or disinterest can act as barriers to effective learning. For example, Esteban et al. (2019) demonstrated a clear correlation between students' emotional expressions prior to microbiology lessons and their subsequent learning outcomes. These findings underscore the need to actively consider the emotional landscape of students, particularly in content‐heavy disciplines like microbiology.

Emotional intelligence (EI), as conceptualised by Goleman and Boyatzis (2017), encompasses self‐awareness, self‐regulation, empathy and teamwork—competencies that are increasingly recognised as essential in both education and professional practice. Within the microbiology classroom, these competencies can be cultivated through team‐based learning, fieldwork, laboratory collaboration and reflective activities. These practices create opportunities for students not only to learn scientific content, but also to understand their own emotional responses, recognise their contributions within a group and manage relationships and responsibilities effectively.

Furthermore, microbiology provides real‐world contexts that can provoke emotional and ethical reflections, such as discussions around vaccination, antibiotic resistance and genetically modified organisms. These situations promote empathy and emotional engagement, which are particularly relevant for future healthcare professionals (Dott et al. 2022). Classroom dialogue around such topics encourages students to confront their assumptions, engage in respectful debate, and develop a sense of ethical and scientific responsibility. This becomes especially important in a society increasingly influenced by misinformation, where a scientifically literate and emotionally intelligent public is vital.

Educators can leverage microbiology's intersection with daily life to stimulate not only cognitive learning but also emotional and ethical awareness. Case‐based learning and problem‐oriented projects offer emotionally resonant and socially relevant narratives that deepen students' understanding of both scientific content and human experience. These discussions challenge students to consider diverse perspectives, weigh societal implications and practise empathy and communication—core components of both emotional intelligence and responsible citizenship.

Ultimately, embedding emotional intelligence into microbiology education not only enhances academic engagement and group collaboration but also prepares students—particularly future healthcare and science professionals—to navigate complex, emotionally charged and ethically nuanced situations in their careers. It empowers them to become not just knowledgeable, but emotionally and socially competent contributors to science and society.

### 3.2 Creativity in Microbiology Education

Young people often perceive science as unengaging and lacking in creativity (Gianotti and Nurse 2023; Valenti et al. 2016). Traditional methods for teaching (micro)biology—such as heavy reliance on textbooks and syllabi focused on memorising taxonomic classifications, rigid and uncritical use of laboratory protocols and the absence of hands‐on activities—can distance students from the subject, stripping it of its more fascinating and dynamic aspects (Hu 2024; Tanner 2013; Tindan and Anaba 2024). To nurture a deeper engagement with microbiology, it is crucial that both teachers and students share a passion for creativity in the learning process. As Noor (2023) aptly noted, *current research in microbiology is pushing the boundaries of our knowledge and paving the way for innovative solutions to some of the most pressing global challenges*. Thus, stimulating reflection, curiosity and creativity is essential for nurturing the next generation of medical doctors, microbiologists, ecologists, biotechnologists and beyond.

Creativity, as a broad competence, encompasses various skills including interdisciplinarity, critical thinking and problem‐solving. It encourages students to question the *status quo*, explore new possibilities and make connections between seemingly unrelated concepts. Consequently, creative students are more inclined to think divergently and *outside the box*, fostering greater confidence and a sense of freedom—allowing them to propose ideas without fear of failure. Several studies highlight the effectiveness of creative teaching approaches in microbiology, utilising various art forms like dance, mime and comics to engage students (Fahnert 2017; Harper et al. 2019; Morel et al. 2019; Rodríguez et al. 2019; Witucki et al. 2018).

For instance, one study demonstrated that undergraduate students participating in an introductory microbiology laboratory course showed increased confidence in their scientific abilities when involved in open‐inquiry activities like agar art, compared to those in a control group (Adkins et al. 2017). This suggests that incorporating visual art into the course‐based undergraduate research experience can significantly enhance student engagement and self‐efficacy. Similarly, Harper et al. (2019) explored integrating dance, poetry and mime into microbiology teaching, offering a creative framework for students to explore microbiological concepts in a more interactive way. In another creative example, Todorova et al. (2015) utilised bacterial cultures on Petri dishes to create coloured images, highlighting how microbiological processes can intersect with artistic expression. Rodríguez et al. (2019) found that flipped classroom methodologies in biology promoted students' perceptions of enhanced creativity, critical thinking and social awareness. Morel et al. (2019) used comics to teach concepts such as antibiotic resistance, further illustrating how creative techniques can be used to deepen student understanding of microbiology. Lastly, an interdisciplinary approach grounded in food fermentation and flavour science has proven effective in engaging students, fostering curiosity and improving their conceptual learning and scientific skills (Sörensen 2023).

In addition to these methods, film forums have been shown to be a valuable pedagogical tool, allowing students to reflect on microbiological concepts through the lens of cinema and fostering critical discussion and creativity (Sánchez‐Angulo, 2023; Linares et al., 2021). Moreover, encouraging students to participate in science fairs—or even organising them—can greatly enhance their communication skills, societal engagement and sense of inclusion, particularly when these fairs are held in underserved schools or communities (e.g., Janštová et al. 2024; Rios‐Velazquez et al. 2006).

Exploring themes such as the discovery of penicillin, the development of vaccines and the use of enzymes in various industries can serve as springboards for student creativity, sparking the development of new ideas and projects within a network of key concepts like *interdisciplinary learning*, *hands‐on experiments* and *problem‐solving skills*. Encouraging these connections nurtures an environment where creativity becomes a driving force in scientific discovery and learning (Table 3).

**TABLE 3:** Examples of SSE strategies and topics in microbiology to promote UNESCO competencies and future skills.

**Exploiting Modern and Complex Scientific Concepts** Exploring advanced microbiological topics within societal and personal contexts promotes competencies such as critical thinking, complex problem solving, emotional intelligence and empathy and creativity. These topics are ideal for problem‐based learning (PBL) and teamwork, also enhancing skills in communication and collaboration.	**Examples of SSE Topics in Microbiology:** Microbial roles and dynamics in various environments (e.g., human, plant, aquatic, and soil ecosystems) Microbe‐microbe and microbe‐host interactions, including the microbiome concept Molecular and biological processes such as replication, metabolism and pathogen‐host interactions Biotechnological applications of microbes (e.g., remediation, fermentation, vaccine production) Biohazards and pathogen transmission, including principles of immunology and vaccination Infectious diseases: diagnosis, treatment and prevention (e.g., COVID‐19, STIs, avian flu) Antibiotic resistance: causes, consequences and mitigation strategies Microbial roles in bioremediation and waste management The human microbiome and its impact on nutrition, health and behaviour Hygiene and public health practices to reduce infections Climate change and microbial ecosystems (e.g., permafrost microbes) Synthetic biology and bioethics CRISPR and gene editing: applications and ethical considerations Probiotics and personalised nutrition Microbial life in space and planetary protection Zoonoses and the One Health approach Microbial degradation of plastics Microbial fuel cells and sustainable energy Fermentation and cultural microbiology Water safety and microbial indicators Forensic microbiology Microbial materials, including self‐healing concrete
**Exploring Practical and Collaborative Work through Case Studies** Hands‐on and case‐based learning encourages critical thinking, problem‐solving, emotional intelligence, empathy, creativity and teamwork. It also supports motor skill development and reinforces scientific inquiry.	**Examples of Practical SSE Approaches:** Culturing microorganisms, staining, microscopy and disinfection comparisons Formulating hypotheses (e.g., *Does baker's yeast tolerate heat better than other strains?*) Designing and conducting experiments Data analysis (e.g., growth curves, replicates) Interpreting results and biological significance Troubleshooting and reflecting on experimental errors Student‐designed projects for ownership and creativity Role‐plays (e.g., a health educator advising a community) Narrative‐based learning (e.g., patient recovery stories to discuss empathy) Regional case studies (e.g., cholera in Haiti, tuberculosis in India, AMR in Africa)
**Valorising the Interdisciplinarity of Microbiology** Linking microbiology to other disciplines fosters integrated learning and helps students see its real‐world relevance.	**Interdisciplinary Connections:** Chemistry: pH, ion exchange, fermentation Physics: microscopy, sterilisation techniques Mathematics: data interpretation, growth rates, infectivity (R_o_), magnification Medicine/Health: immunology, disinfection, One Health Food Science: fermentation, food safety, gut microbiota Biotechnology: vaccines, antimicrobials, genetically modified organisms (GMOs) Agriculture, farming and soil science Environmental Science: biogeochemical cycles, bioremediation Digital/Computer Science: bioinformatics, simulations, citizen science Bioengineering and sensors Energy: biofuels, bioelectricity Geology and geomicrobiology Meteorology Construction and the built environment: material deterioration/protection Social Sciences: behavioural health, cultural practices, public response to pandemics Law and justice: forensics Art: creative communication of microbiological concepts History: pandemics over time Paleomicrobiology Economics: impact of AMR on healthcare systems
**Using Digital and Media Resources for Critical Thinking** Incorporating digital tools and resources builds students' capacity to critically evaluate information and fosters societal engagement.	**SSE Strategies Using Media:** Evaluate scientific papers and news for credibility and bias Analyse conflicting data and scientific debates Compare social media with credible sources on microbiology issues Structured debates (e.g., CRISPR, vaccine mandates) Bioethics discussions (e.g., vaccine distribution) Public health communication projects (e.g., hygiene campaigns) Create infographics or videos for diverse audiences
**Innovative Learning Strategies to Promote Creativity** Microbiology can be explored creatively even with minimal resources.	**Innovation‐Promoting Strategies:** Low‐cost experiments (e.g., fermentation, handwashing demonstrations) Virtual labs and open‐source simulations Storytelling, role‐play and debates Local or household mini projects (e.g., tracking spoilage or GMOs in food labels) Cross‐curricular projects (e.g., linking health and geography) Citizen science (e.g., mould mapping, hygiene audits) PBL scenarios (e.g., *Escherichia coli* outbreak response, do‐it‐yourself (DIY) water testing kits) Creative contests (poetry, comics, video challenges) Film forums (e.g., microbiology in movies to promote critical reflection and discussion) Hackathons or innovation sprints (e.g., sustainable biotech ideas) Maker activities (e.g., microbial fuel cells, wild yeast fermentation, Winogradsky columns) Participation in science fairs or their organization (e.g., showcasing microbiology projects to enhance communication and societal engagement) Teacher‐friendly modules and open‐access resources for complex topics
**Promoting Inclusion and Diversity** Embedding microbiology in diverse cultural contexts enhances engagement and relevance.	**Inclusion Strategies:** Culturally responsive curriculum with global case studies Showcase microbiologists from diverse backgrounds Multilingual and accessible materials (e.g., subtitles, mobile‐compatible tools)
**Embedding Ethical and Societal Questions** Ethics discussions make microbiology relevant to civic life and personal values.	**Ethics‐Focused Strategies:** Debates on gain‐of‐function research, CRISPR and global antibiotic access Case‐based discussions on moral dilemmas in science Science communication projects (e.g., vaccine awareness) Student outreach partnerships with local health organisations Service‐Learning projects focused on disadvantaged populations Discussions on the importance of activities (such as Service‐Learning) and organisations (such as Médecins Sans Frontiers) created to help the disadvantaged
**Encouraging Inclusive Group Work** Diverse group work fosters equity and collaborative skills.	**Collaborative Learning Approaches:** Team‐based learning with rotating roles (e.g., leader, researcher, presenter) Encouraging active participation from all students Promoting mutual respect in group dynamics

### 3.3 Nurturing Critical Thinking and Complex Problem Solving in Microbiology Education

In an era of rapid scientific advancement, microbiology education must evolve beyond rote memorization and procedural routines. Central to this transformation is the cultivation of critical thinking—the ability to systematically analyse, evaluate and synthesise concepts, models and data, and to seek relevant information and evidence‐based discussion, opinion‐formation and decisions. In microbiology, this involves questioning assumptions, identifying strengths and weaknesses in scientific arguments and integrating multiple sources of knowledge to form coherent, evidence‐based conclusions (Aminuddin and Baitun 2017). One approach to foster critical thinking in general, using examples from microbiology, is storytelling based on discussions of relevant issues (Timmis et al. 2024), such as correlation and causality, bottlenecks/rate limiting parameters (e.g., IMiLI 2024).

Critical thinking in microbiology can be fostered by encouraging students to analyse and even co‐create foundational concepts such as the distinction between prokaryotic and eukaryotic cells, cellular morphology, organelle functions, cell division processes, metabolic pathways like glycolysis and fermentation, bacterial growth dynamics and regulatory or environmentally influenced phenomena such as facultative pathogenicity, quorum sensing and microaerophily. It is crucial to cultivate a mindset of perpetual questioning, constantly asking *why?* These topics are not only central to the discipline but also provide fertile ground for interpretation, comparison and conceptual modelling—skills that deepen scientific literacy and comprehension. Moreover, microbiology is a dynamic field marked by continuous discovery and evolving interpretations. Students must therefore be equipped to navigate scientific uncertainty, critique emerging hypotheses and draw informed conclusions from primary literature and expert opinion. This reflective capacity empowers them to engage meaningfully in contemporary scientific discourse.

Equally vital is the development of systems thinking and complex problem‐solving skills. These competencies require students to apply foundational microbiological knowledge in practical, often interdisciplinary, contexts. Complexity can be scaled according to students' educational level and background, progressing from basic scenarios to intricate, real‐world challenges. Inquiry‐driven approaches such as project‐based and problem‐based learning are particularly effective in this regard. For instance, students might investigate how enzymes are employed in biotechnological processes, understand the steps behind generating genetically modified organisms, explore immunological responses at the molecular level or analyse the determinants of herd immunity and the nexus between vaccination, immune responses, human behaviour, mathematics and modelling. These explorations not only reinforce core concepts but also encourage students to draw connections across domains—integrating knowledge from genetics, biochemistry, immunology, bioinformatics and bioethics.

Another essential dimension involves examining microbiological risk, safety and ethics. By critically analysing biosafety guidelines and bio risk management practices, students confront real‐world dilemmas, requiring them to evaluate the rationale and implications of safety protocols. This reflection not only enhances laboratory responsibility but also strengthens decision‐making in complex, ambiguous contexts.

Microbiology inherently supports both critical thinking and problem‐solving through the application of the scientific method. This includes:

- Formulating testable hypotheses and research questions,
- Designing experiments that appropriately test these hypotheses,
- Collecting, analysing and interpreting data, often with the aid of statistical and digital tools, and
- Drawing conclusions that either support or refute the initial hypothesis.

Such experiences emphasise that science is not static knowledge but a process of iterative questioning, validation and refinement. Students learn that understanding in microbiology—much like the field itself—is provisional, contextual and open to reinterpretation considering new data. They are encouraged to incorporate historical perspectives, weigh ethical implications and appreciate the evolving nature of scientific knowledge.

### 3.4 Collaborative Work and Leadership in Microbiology Education

Collaboration is a cornerstone of scientific practice, and microbiology education offers rich opportunities to cultivate teamwork and leadership skills among students. Microbiology projects often require joint effort—whether through laboratory experiments, fieldwork or research assignments—encouraging students to share ideas, challenge assumptions and co‐create solutions in a collective environment. This dynamic fosters not only academic growth but also essential interpersonal competencies that are foundational for future scientific and professional success.

A variety of instructional strategies can be employed to leverage collaboration. These may include team‐based research on microbiological topics, cooperative design and execution of laboratory experiments or the analysis of real‐world case studies—such as infectious disease outbreaks, diagnostic protocols or antibiotic resistance scenarios. These tasks challenge students to apply scientific knowledge in practical contexts while relying on one another's strengths, promoting both innovation and diversity of thought.

Collaborative environments offer more than just academic benefits. As students work together toward shared goals, they learn to build relationships grounded in mutual respect, empathy and trust—core elements of successful teamwork (UNESCO 2016). This social interaction strengthens classroom engagement and motivation, creating a more inclusive and participatory learning atmosphere. Students also develop communication skills by articulating their ideas, actively listening to others and engaging in respectful, evidence‐based discussion. For example, structured peer discussions around contemporary microbiological questions—such as *How does the gut microbiome influence health?*, *Why are vaccines vital for public health?*, *What are bacteriophages and how can they help combat bacterial pathogens?* or *what drives the layering seen in a Winogradsky column?*—encourage students to synthesise information, reason through complex issues and build persuasive arguments rooted in scientific facts. These conversations mirror the collaborative discourse that defines the scientific community, thereby preparing students for authentic scientific engagement.

Leadership development can be naturally integrated into collaborative learning environments. Rotating leadership roles—such as assigning spokespersons, project coordinators or lab team leads—allow students to practice organising group tasks, mediating differing perspectives and managing expectations. Furthermore, problem‐based and project‐based teaching methods amplify the benefits of collaboration. These pedagogies challenge students to investigate real‐world microbiological problems, divide responsibilities and co‐develop and present comprehensive solutions. This, combined with promoting a culture of constructive questioning of the rationale behind opinions, statements and decisions (a *why?* culture) helps develop critical thinking, problem‐solving skills and maturation of leadership qualities. As a consequence, students develop confidence and gain essential skills in coordination, empathy and conflict resolution (Darling‐Hammond et al. 2020; McInerney and Fink 2003; Traini et al. 2025) which enhance their capacity for collaboration in diverse team settings.

Ultimately, collaborative learning in microbiology education enriches both cognitive and social–emotional development. It allows students to engage more deeply with the subject matter, promotes open‐mindedness and scientific reasoning, and equips them with transferable skills vital across disciplines and professions. By learning how to navigate teamwork, manage conflict, and lead with purpose, students are better prepared to contribute meaningfully to the scientific and broader global community.

### 3.5 Efficient Communication in Microbiology Education—Building Literacy and Societal Engagement

In an era defined by rapid scientific advancement and widespread misinformation, the role of microbiology education extends far beyond the classroom. One of its most critical dimensions lies in equipping students—and by extension, future citizens—with the ability to communicate scientific knowledge effectively. This includes not only internal communication within scientific and academic communities, but also outward communication to society, which is vital for public acceptance of new technologies, but where science must contend with misunderstanding, scepticism, and misinformation.

Microbiology offers a unique platform for developing these communication skills. By exploring the diverse roles of microbes—beneficial, neutral and harmful—students begin to see the relevance of microbiological knowledge in everyday contexts, from healthcare and food safety to climate science and biotechnology. This relevance fosters deeper engagement and empowers students to articulate complex concepts clearly and accurately, both verbally and in writing.

Effective science communication involves two fundamental dimensions: the ability to express scientific ideas clearly and persuasively, and the ability to listen and respond thoughtfully in dialogue with others (UNESCO 2016). In microbiology, this requires cognitive flexibility—linking ideas, interpreting scientific data and arguing for or against concepts using scientific language and evidence. It also requires emotional intelligence, especially when discussing ethically or socially charged topics such as vaccination, antibiotic resistance or the use of genetically modified organisms.

In this regard, art and creativity can serve as powerful tools. As explored in the creativity dimension of microbiology education, incorporating artistic expressions—such as visual media, storytelling and performance—can make abstract or technical content more accessible and engaging. These methods not only enhance comprehension but also promote public interest and dialogue, further bridging the gap between science and society. A particularly urgent challenge is the prevalence of misinformation, especially in online environments. With young people being the primary users of social media platforms, microbiology education must go beyond simply transmitting knowledge. It must empower students to critically evaluate the information they encounter, identify misinformation and become active sharers of credible scientific data. Educational strategies can include social media‐based projects where students create content to explain scientific concepts, dispel common myths or respond to trending misinformation with evidence‐based posts.

Moreover, microbiology education should promote stewardship awareness and social responsibility—global citizenship (UNESCO 2023) by encouraging students to explore the societal implications of microbiological knowledge (Timmis et al. 2024). When students engage in projects that aim to solve real‐world problems—such as tackling antimicrobial resistance or improving public hygiene—they develop a stronger sense of belonging to and responsibility for their communities. This strengthens the link between individual learning and collective well‐being, positioning science as a tool for societal advancement.

Educational practices that support this mission may include collaborative presentations, research reports and structured classroom debates on controversial or emerging topics. For example, students might debate the ethics of gene editing or the global implications of vaccine access. These activities not only reinforce scientific knowledge but also cultivate persuasive communication, critical listening and the ability to engage respectfully with differing perspectives.

Ultimately, microbiology education that prioritises efficient communication nurtures scientific literacy, enhances student engagement and strengthens the connection between science and society. By developing articulate, thoughtful communicators who can navigate and contribute to informed public discourse, we lay the foundation for a more scientifically literate and resilient society.

## 4 Take‐Home Message and Future Perspectives

Microbiology education has the power to transform how students understand and interact with the world around them. By linking microbiological concepts to real‐life issues—such as food production, health and environmental sustainability—it becomes more relevant, engaging and impactful. These connections not only enhance learning outcomes but also feed open dialogue and critical communication skills, empowering students to think scientifically and act responsibly in society.

Innovative educational approaches such as interdisciplinary‐learning and active learning promote deeper student engagement by encouraging curiosity, discovery and peer‐to‐peer communication. When students actively participate in constructing and sharing knowledge, they become more confident, reflective and capable of applying their learning to complex, real‐world challenges.

However, as technology becomes increasingly integrated into educational methods, issues of social equity persist. While digital tools can democratise access to information, disparities in internet access and digital literacy—especially among students from low‐income or under‐resourced communities—highlight the need for inclusive educational strategies. These challenges call for collaborative efforts from educators, policymakers and communities to ensure that all students can benefit equally from modern microbiology education.

Looking forward, future perspectives should emphasise:

– Strengthening the integration of microbiology with societal and environmental issues.
– Expanding the use of interdisciplinary and creative teaching methods.
– Promoting equitable access to educational technologies and digital infrastructure.
– Cultivating a culture of communication, critical thinking and civic responsibility among students.

In this context, the proposed strategies could also inspire university teachers and researchers (who tutor PhD students) to implement them in their teaching practices, engage students in developing additional competencies, and contribute to shaping the ‘informed, responsible, and engaged global citizens’ highlighted in the concluding sentence of this manuscript.

In doing so, microbiology education will not only prepare students to thrive academically and professionally but also equip them to be informed, responsible and engaged global citizens.

## Author Contributions

**Lara Amorim:** conceptualization, writing – original draft, investigation, visualization. **Conceição Santos:** validation, conceptualization, writing – review and editing, supervision. **Kenneth Timmis:** writing – review and editing, validation.

## Conflicts of Interest

The authors declare no conflicts of interest.

## Data Availability

Data sharing not applicable to this article as no datasets were generated or analysed during the current study.
